# The effect of a pre- and post-operative orthogeriatric service on cognitive function in patients with hip fracture. The protocol of the Oslo Orthogeriatrics Trial

**DOI:** 10.1186/1471-2318-12-36

**Published:** 2012-07-20

**Authors:** Torgeir Bruun Wyller, Leiv Otto Watne, Anne Torbergsen, Knut Engedal, Frede Frihagen, Vibeke Juliebø, Ingvild Saltvedt, Eva Skovlund, Johan Ræder, Simon Conroy

**Affiliations:** 1 Institute of Clinical Medicine, University of Oslo, Oslo, Norway; 2 Department of Geriatric Medicine, Oslo University Hospital, PO Box 4956 Nydalen NO-0424, Oslo, Norway; 3 Norwegian Centre for Ageing and Health, Oslo, Norway; 4 Orthopedic department, Oslo University Hospital, Oslo, Norway; 5 Department of Medicine, Akershus University Hospital, Lørenskog, Norway; 6 Department of Geriatrics, St. Olav Hospital, University Hospital of Trondheim, Trondheim, Norway; 7 Department of Neuroscience, Norwegian University of Science and Technology, (NTNU), Trondheim, Norway; 8 School of Pharmacy, University of Oslo, Oslo, Norway; 9 Department of Anaesthesiology, Oslo University Hospital, Oslo, Norway; 10 Geriatric Medicine, Department of Cardiovascular Sciences, University of Leicester School of Medicine, Leicester, UK

## Abstract

**Background:**

Hip fractures mainly affect older people. It is associated with high morbidity and mortality, and in particular a high frequency of delirium. Incident delirium following hip fracture is associated with an increased risk of dementia in the following months, but it is still not firmly established whether this is an association or a causal relationship. Orthogeriatric units vary with respect to content and timing of the intervention. One main effect of orthogeriatric care may be the prevention of delirium, especially if preoperative and postoperative care are provided. Thus, the aim of Oslo Orthogeriatric Trial, is to assess whether combined preoperative and postoperative orthogeriatric care can reduce the incidence of delirium and improve cognition following hip fracture.

**Methods/design:**

Inclusion and randomisation will take place in the Emergency Department, as soon as possible after admission. All patients with proximal femur fractures are eligible, irrespective of age, pre-fracture function and accommodation, except if the fracture is caused by a high energy trauma or the patient is terminally ill. The intervention is pre-and post-operative orthogeriatric care delivered on a dedicated acute geriatric ward. The primary outcome measure is a composite endpoint combining the Clinical Dementia Rating Scale (CDR) and the 10 word memory task at four months after surgery. Secondary outcomes comprise incident delirium, length of stay, cognition, mobility, place of residence, activities of daily living and mortality, measured at 4 and 12 months after surgery. We have included 332 patients in the period 17^th^ September 2009 to 5^th^ January 2012.

**Discussion:**

Our choice of outcome measures and our emphasis of orthogeriatric care in the preoperative as well as the postoperative phase will enable us to provide new knowledge on the impact of orthogeriatric care on cognition.

**Trials registration:**

ClinicalTrials.gov NCT01009268

## Background

### Falls and hip fracture in older people

Approximately 30% of home-dwelling people aged 65+ fall each year, and half of them fall twice or more. Ten percent of the falls result in serious injuries [1]. Multimorbidity and frailty often complicate the assessment of falls amongst older people. Scandinavian countries have the highest reported incidence of hip fractures (cervical or trochanteric) in the world [2], 90% of which are related to a fall. Hip fractures are associated with considerable mortality [3,4]. Due to the increasing number of older people, the demand for treatment, rehabilitation and care for hip fracture patients will increase steeply during the coming years. Older people with hip fracture after a fall have more comorbidity, dementia and physical disability than older people who fall without developing a fracture [5].

In a retrospective study carried out among patients surviving a hip fracture at Ullevaal University Hospital, the one-year mortality was 23%, and the number of institutionalised patients rose from 15% before the fracture to 30% after one year. The number of patients dependent on a walking aid increased from 35% before the fracture to 76% after one year, 43% of the patients had lost the ability to walk independently outdoors, and 25% had lost the ability to prepare food independently [6].

### Delirium as a complication of hip fracture

In a retrospective cohort study of 364 patients with hip fracture, the prevalence of delirium on admission was 21.1%, and the incidence of postoperative delirium among those who were non-delirious **pre**operatively was 36.4% [7]. Amongst those without evidence of any cognitive decline before the fracture (measured using the Informant Questionnaire on Cognitive Decline in the Elderly (IQCODE) [8], the risk of dementia at six months was strongly associated with perioperative delirium, suggesting a possible causal relationship between delirium and the development of dementia [9], in keeping with several other studies [10-12]. Accordingly, an attractive hypothesis might be that preventing delirium in the acute phase of hip fracture may also prevent a rapid development of chronic cognitive failure or dementia in these patients. This hypothesis can, however, only be tested by an experimental design.

### Orthogeriatrics – a complex intervention for patients with hip fracture

Orthogeriatrics describes various forms of structured cooperation between orthopaedic surgeons and a multiprofessional geriatric team [13-16]. The 2009 Cochrane review included 13 trials, most with some form of geriatric input into the acute or sub-acute postoperative phase of hip fracture, [17]. The studies were heterogeneous in terms of the interventions and the outcomes assessed, but indicated a non-significant trend towards a lower risk of a poor outcome with the intervention (risk ratio 0.89, 95 % confidence interval (CI) 0.78-1.01). Kammerlander *et al*[18], categorised orthogeriatric care into four models, described by Pioli [14]:

1. Orthopaedic ward and geriatric consultant service.

2. Orthopaedic ward and daily consultative service.

3. Geriatric and rehabilitation ward and orthopaedic consultant service.

4. Orthopaedic ward and integrated care.

They found no clear evidence in favour of any particular model, though there was a trend in favour of models based upon strong integration of the orthopaedic and the geriatric service. A very recent trial found no effect of an intervention that only comprised a liaison team [19]. Recent NICE guidelines for treatment of hip fracture patients recommend early orthogeriatric involvement, but give no clear recommendation with respect to how this should be organised [20].

A few studies have explicitly evaluated the possible effect of orthogeriatric care upon the risk of delirium. A Spanish single blinded, randomised controlled study evaluated the effect of a geriatric consultative team giving mainly medical input within a conventional orthopaedic ward. There was no effect on length of stay (the primary endpoint), but a pronounced effect upon postoperative complications, mainly delirium (34% versus 44%) and pressure sores (5% versus 17%) [21].

One randomised, controlled study from Umeå (Sweden), evaluated the effect of a specialised orthogeriatric ward, only for postoperative treatment and early rehabilitation. In comparison with patients cared for in ordinary orthopaedic wards, the intervention patients suffered less postoperative delirium (58% versus 76%) and the duration of delirium was shorter (2.7 versus 7.7 days). There was also a statistically significant reduction in the risk of other complications as well as in length of stay. However, the risk of preoperative delirium was high and without significant between-group differences [22].

We have only identified nine published studies that explicitly evaluated orthogeriatric wards for *combined preoperative and postoperative* treatment. Most of them suffer from methodological problems, but in sum they suggest a possibly better effect of this organisational model than from a purely post-operative intervention.

At the Piteå River Valley Hospital in Sweden, the treatment results were compared with those of historical controls from the same hospital as well as other hospitals in Sweden and internationally. The intervention was associated with an increased likelihood of returning to independent living (89% vs. 58-60% in the different control groups), and a reduced risk of preoperative (20% vs. 29-33%) and postoperative (31% versus 48-61%) delirium [23].

In an American study, Marcantonio *et al* randomised 126 hip fracture patients to either care as usual or proactive geriatric consultation on the orthopaedic ward, starting preoperatively when feasible [24]. Delirium occurred in 32% of the intervention patients versus 50% of the usual-care patients (relative risk 0.64, 95% CI 0.37-0.98). In another American study, Friedman and co-workers established a Geriatric Fracture Centre with geriatricians and orthopaedic surgeons working closely together in the preoperative as well as in the early postoperative period. The risk of delirium as well as other complications was lower amongst patients treated within this model compared to historical controls [25] as well as to a national register [26].

In Tel-Aviv (Israel), the allocation between an orthogeriatric ward (for preoperative and postoperative treatment) and ordinary orthopaedic wards occurred on a non-randomised basis [27,28]. When adjusted for prognostically important variables, the odds ratio for a successful rehabilitation was 1.97 (95% CI 1.09 - 3.65) in favour of the intervention.

Swanson and co-workers carried out a small randomised controlled trial (RCT) in Australia, including only patients that were independently mobile prior to the fracture, and provided early surgery, minimal narcotic analgesia, intense daily therapy and a multidisciplinary approach preoperatively as well as postoperatively. They found a shorter length of stay (LOS) in the intervention group, but did not report on delirium or other cognitive end-points [29].

In a study from Taiwan comprising 137 patients selected on the basis of good cognitive and physical prefracture function, the authors reported better function in walking performance and activities of daily living (ADL), fewer falls and fewer depressive symptoms in patients who were offered interdisciplinary geriatric assessment in the preoperative as well as in the postoperative phase [30], and the effect lasted for at least two years [31]. The risk of delirium or other cognitive effects were not reported.

Zuckerman and co-workers [32] reported an interdisciplinary geriatric care program for hip fracture patients, compared to historical controls. They found fewer complications, better ambulatory ability and a reduced need for nursing home care among those cared for in the program, but do not report effects on delirium or cognition.

Koval and co-workers [33] compared patients treated using an integrated and multiprofessional pathway, comprising preoperative as well as postoperative elements, with historical controls. They found a decrease in length of stay and mortality. Another study of a geriatric team in an orthopaedic department, compared to historical controls, found no change in length of stay but a decrease in the risk of postoperative complications [34].

Thus, there is some evidence that orthogeriatric services are effective, and that one of the effects may be a reduced risk of delirium. There is some evidence that .effectiveness is greater if the hip fracture patients are admitted directly to a specialised orthogeriatric ward were they are stabilised, treated and rehabilitated in the preoperative as well as the early postoperative phase. Delirium in hip fracture patients usually starts preoperatively and last into the postoperative phase [7], accordingly, efforts to prevent delirium must start preoperatively.

The aim of this study was to robustly evaluate an interdisciplinary model of orthogeriatric care combining pre and post-operative intervention, using cognition as the outcome.

### Study objectives

#### Primary objective

· To assess the effect upon cognitive performance four months after surgery of a model of preoperative as well as early postoperative care, treatment and rehabilitation of hip fracture patients in a dedicated orthogeriatric unit compared to treatment as usual in a conventional orthopaedic ward.

#### Secondary objectives

· To estimate the effect of the intervention upon the incidence of delirium during the acute stay, and on length of stay.

· To estimate the effect of the intervention upon place of residence, activities of daily living, cognition, mobility and mortality, measured at 4 and 12 months after surgery.

· To study differences in mobilisation practices between the two wards.

## Methods/design

### Project context

Based upon the results of a pilot study [35], Ullevaal University Hospital (now part of Oslo University Hospital) started a reorganisation of the treatment of hip fracture patients in 2008, in line with the best available evidence. We organised an orthogeriatric service as a part of the acute geriatric ward during the autumn of 2008 and spring of 2009. Clinical procedures and routines for the service as well as the cooperation between the geriatricians and the orthopaedic surgeons were piloted. However, the capacity of the new orthogeriatric unit was insufficient for the catchment area of the hospital, and we thus decided to allocate patients randomly between the new and the traditional ward in order to generate valid new evidence from a situation characterised by scarce hospital beds. Randomisation started on September 17th, 2009.

The study is carried out in close cooperation between the Department of Orthopaedic Surgery and the Department of Geriatric Medicine. The catchment area for the hospital with respect to orthopaedic trauma has changed slightly during the project period, and in 2011 consists of five local municipalities with a total population of about 200,000. All patients admitted to the Emergency department with a hip fracture are assessed for eligibility.

### Study design

The study is a randomised, controlled, single-blind study comparing preoperative and postoperative orthogeriatric care integrated in the acute geriatric ward, to care as usual in the orthopaedic ward. Recruitment and randomisation take place in the Emergency Department.

### Study population

Eligible patients are those admitted acutely to Ullevaal University Hospital (now: the Ullevaal Clinic of Oslo University Hospital) for a femoral neck fracture, a trochanteric or a sub-trochanteric femoral fracture as result of a low energy trauma, defined as fall from own height or from a level not higher than 1 metre.

Patients are excluded if the hip fracture is part of multi-trauma or high energy trauma (defined as a fall from a higher level than 1 metre). One recent fracture in addition to the hip fracture (e.g. radius or shoulder) is acceptable. We also exclude patients who are regarded as moribund at admittance (as judged by the admitting orthopaedic surgeon) and patients lacking a valid informed consent or assent.

There are no exclusion criteria related to age. Most younger (below 70 years) hip fracture patients have suffered a high energy trauma, and thus are excluded. The small number of patients of this age that suffer a hip fracture from a low energy trauma, are expected to be frail and thus potentially benefit from an orthogeriatric service. We also include patients who fracture their hip when already permanently institutionalised in a nursing home. We believe that this particularly frail group may benefit as much as more fit individuals from this type of service.

Some patients initially admitted to the Ullevaal Clinic, are sent to other hospitals in Oslo for surgery due to capacity problems in the operation theatre, and then back again for further care within a few hours postoperatively. Such patients are eligible, and are randomised to receive either the intervention or the usual care preoperatively as well as postoperatively. On the other side, patients are not eligible if they come from other nations or from other parts of the country, fracturing their hip during a visit to Oslo, and thus are sent to a hospital at their domicile shortly after surgery.

Some eligible patients have not been included due to surgical procedure failure. Patients in whom the diagnosis is uncertain at admittance have been admitted to the orthopaedic ward whilst waiting for further diagnostic procedures (CT or MR of the hip), and thus not included. Inclusion of patients has also been put on hold in periods where intake to the acute geriatric ward is closed due to uncontrolled outbreaks of infections (mostly Noro virus). A Refused, Missed or Otherwise excluded (RMO) database is maintained to determine the feasibility of a larger study and to assess the generalisability of the enrolled sample to all cases.

### Intervention

#### Common elements

All patients with a suspected hip fracture are assessed in the Emergency Department by the orthopaedic resident on call, who establishes the diagnosis and notifies the Operating Theatre about the patient. The patients undergo surgery according to the established routine of the orthopaedic department, which has remained unchanged throughout the study period. Patients with trochanteric fractures are operated with a sliding screw device, undisplaced femoral neck fractures with two parallel screws, and displaced femoral neck fractures with arthroplasty, mainly bipolar hemiarthroplasty [36]. All receive prophylaxis against thromboembolism by the use of dalteparin (2500 IU two times daily preoperatively, and 5000 IU once daily after the operation and until fully mobilised). All also receive peroperative antibiotic prophylaxis with cephalotin .

Patients allocated to the intervention group are transferred as soon as possible to the acute geriatric ward, stabilised preoperatively, and transferred back to the same ward following surgery for further treatment and rehabilitation after a short stay in the recovery unit for immediate postoperative stabilisation. The control patients are treated in the orthopaedic ward preoperatively and after the recovery unit stay. The stay in the recovery unit regularly lasts for a few hours, but occasionally the patients may stay in the recovery unit or the intensive care unit for several days if life-threatening perioperative or early post-operative complications occur. The orthopaedic surgeons examine patients in the acute geriatric ward upon request, which is reciprocated by the geriatricians for patients in the orthopaedic ward. The intervention is not designed to impact upon time to surgery. The two departments are located in different buildings, but the buildings are connected with each other as well as with the Emergency Department, the Operating Theatre, and the Radiology Department with in-door paths.

#### Experimental group

The Department of Geriatric Medicine runs a 20 bed-ward mainly admitting patients suffering from acute medical disorders superimposed upon frailty, co-morbidities and polypharmacy, many of whom suffer from or are at high risk of delirium. Only hip fracture patients that participate in this study are admitted to the acute geriatric ward, and for most of the project period three to four of the beds are used for hip fracture patients. Most of the time the acute geriatric ward is full or over-crowded, implying that the hip fracture patients are cared for wherever in the ward a free bed is available, sometimes in the corridor. The ward staffing is 1.33 nurses or nurse assistants per bed, and one physiotherapist and one occupational therapist per 10 beds. A nutritionist is available on request. The orthogeriatric model accords with type 3 according to Pioli's [14] and Kammerlander's [18] categorisation as described above.

Clinical routines for the orthogeriatric service have been developed during the pilot phase in 2008 and 2009, and are documented in the electronic library of clinical handbooks at Department of Geriatric Medicine. The main elements of the intervention are as follows:

· Comprehensive geriatric assessment [37] as a basis for the planning of further measures. All team members (physician, nurse, occupational therapist and physiotherapist) are expected to assess the patients the first day in the ward (some parts of the assessment may be postponed to the first postoperative day), and the team has daily short meetings in order to co-ordinate assessment, treatment and rehabilitation. Relatives and local health authorities are contacted in order to ascertain pre-fracture status. Pre-fracture cognitive decline is assessed by the IQCODE [8], ADL performance (pre-fracture and during the stay) by the Barthel Index [38], delirium symptoms by the Confusion Assessment Method CAM [39], and the Mini Mental State Evaluation (MMSE) [40] is used as a general cognitive screening.

· Prompt and intensive correction of physiological disturbances preoperatively and postoperatively (hypoxemia, anaemia, electrolyte disturbances, acid–base disturbances, dehydration, hypotension etc), based upon pre-defined protocols.

· Tight blood sugar control, with insulin infusion if needed, based upon pre-defined protocols.

· Active optimising of all relevant comorbid conditions.

· Immediate review of all medication and optimisation of the drug regimen.

· Optimal use of analgesics, namely paracetamol 3 g per day supplemented with oxycodone as needed. Nurses and physiotherapists use a numerical rating scale to assess pain during rest and during exercise.

· Repeated short teaching courses for the nursing staff in the non-pharmacological prevention of delirium, emphasising repeated and simple information, use of close relatives, and calming techniques.

· Active monitoring of symptoms of delirium, followed by pharmacological and non-pharmacological treatments as appropriate, as recommended in the recent NICE guidelines [41].

· Prescription of drugs for prophylaxis of further bone loss if indicated.

· Early and intensive mobilisation, supervised by physiotherapists. The patients should be actively mobilised on the first postoperative day, unless clearly contra-indicated [20].

· Early discharge planning and contact with close relatives and the local health authorities.

· Active prevention of prolonged preoperative fasting by the use of two nutrition protein enriched drinks (2x200ml) daily, self-selected food for the meals, use of water, lemonade and carbohydrate enriched drinks until two hours before surgery and normal diet until six hours before surgery.

· Postoperative nutrition intervention by protein enriched meals (35 kcal/kg body weight) during the hospital stay, two nutritional and protein drinks daily (2x 200 ml), vitamin supplementation with 75 μg vitamin K, 5 μg vitamin D and 500 mg calcium (only vitamin D and calcium for patients using oral anticoagulants), and 5 ml cod liver oil or two cod liver oil capsules daily.

· At discharge, the patients are advised to continue to take vitamin supplementation, cod liver oil and protein and energy enriched drinks as described above (and given requisition for this). Individual advice on food preparation, shopping and dental prostheses are also provided, but no specific nutritional intervention take place after discharge.

#### Control group

Control patients receive traditional treatment at the orthopaedic ward. The Department of Orthopaedic Surgery has a 52 bed-ward admitting all kinds of elective as well as acute orthopaedic patients. The staffing ratio is 1.29 nurses or nurse assistants per bed. The amount of physiotherapy is of the same magnitude as on the acute geriatric ward. There is, however, no occupational therapist or nutritionist affiliated to the orthopaedic ward, no regular multiprofessional meetings, and no geriatric assessment as routine.

There is an emphasis on early mobilisation; patients receive physiotherapy regularly, in most instances on every weekday. The nurses have a strong emphasis on mobilisation and nutrition. There are no firm routines for the management of geriatric or internal medical problems in the patients, but relevant specialists see patients on request. The use of analgesics is based on the same principles as in the geriatric ward, but with a less tight follow-up of the treatment effect.

### Primary endpoint

We will construct a composite endpoint using two instruments:

· Clinical Dementia Rating Scale (CDR) [42], is a scale measuring severity of dementia. The scale consists of six questions, each rated 0–3, adding up to a sumscore of 0–18 ("sum of boxes"), or a categorical score between zero and 3. We plan to use the "sum of boxes" scoring. The correlation between the two scoring systems is high, approximately 0.9 [43,44]. The scale is frequently used in dementia treatment trials, and has been shown to be valid and reliable [45]. CDR is scored based on the best available sources, as a combination of patient and proxy information (relative, staff in nursing home or home nursing service).

· The 10 words memory task from the Consortium to Establish a Registry for Alzheimer's Disease battery (CERAD) [46]. This test is shown to be sensitive for memory changes in persons that have a good or fairly good cognitive functioning. We will use the immediate recall and the delayed recall parts of this task, i.e. two scales.

We will normalise these three scales into a 0–100 scoring (and reverse the CDR scoring as this is scaled in the opposite direction) to compute the composite endpoint. Before the randomisation allocation variable is added into the database, we will examine the proportion of patients in the lower *vs* the higher spectrum of performance as well as the correlation between each pair of scales. This information will be used to choose an appropriate weight to each of the scales before combining them. The distribution of the composite endpoint will also be examined.

The primary endpoint is assessed after four and twelve months, by a trained research assistant blinded to allocation.

### Secondary endpoints

· CDR and 10 words memory task analysed seperately

· Preoperative and postoperative delirium, ascertained using the CAM. CAM is a short screening instrument that is validated against the ICD-IV-criteria for delirium, [39] and principally dichotomises the patients in two groups, with and without delirium. A third group, those suffering from sub-syndromal delirium can, however, also be defined. These patients fulfil some, but not all the diagnostic criteria for delirium. The patients are screened on a daily basis (weekdays) for five days after the operation or until discharge by a doctor trained in delirium assessment or one of two study nurses, closely supervised by the study physician. The agreement between the doctor’s and the study nurses’ scorings has been evaluated and found to be very good. In a few cases of missing data in the CAM scores, we have retrospectively constructed the scores based on information from the patient records if relevant information has been available there.

· Duration and severity of delirium (according to the Memorial Delirium Assessment Scale - MDAS) [47]. Initially, MDAS was scored only for patients with at least one fulfilled CAM criterion (sub-syndromal or full delirium), but since July 2011 we have decided to score MDAS in all the patients.

· Incidence of dementia 12 months postoperatively (ICD-10-criteria for research). The diagnosis of incident dementia (as well as that of pre-fracture dementia) will be based upon consensus in an expert panel consisting of one experienced old age psychiatrist (KE) and one experienced geriatrician (TBW), who will have access to all clinical information and results of the cognitive tests, but are blinded to allocation. In the case that no consensus is reached, a third expert will be consulted. We have utilised a similar procedure in a recent project [9].

· Results of other cognitive tests 4 and 12 months postoperatively (MMSE score, clock drawing test score).

· Length of hospital stay, based on patient records.

· Intra-hospital mortality and cumulative mortality at 4, 6 and 12 months postoperatively. Causes of death will be ascertained from the Cause of Death Register, which is coded according to ICD-10.

· Residential status at 4 and 12 months and number of days in own home during the first four months. This is based upon best available information from patient, family, or the local municipality (computerised information held by the local health authorities, and registered in the same way by all municipalities in Norway in the data program Gerica).

· The Barthel ADL Index [38], a ten items index of primary activities of daily living (eating, grooming, toileting etc.), adding up to a sumscore ranging from 0 to 20. The Barthel Index is sensitive to differences among persons with severe or moderate disabilities (but has a profound ceiling effect). The scoring will be based upon proxy information from the best available source (relative, nurse etc). The Barthel Index will be scored at 4 and 12 months postoperatively.

· The Nottingham Extended ADL Index (NEADL) [48], assessing instrumental ADLs like handling money and using public transportation, adding up to a sumscore of maximum 66, and having good sensitivity in the upper part of the functional range, where the Barthel Index functions poorly. Scoring is based upon proxy information 4 and 12 months postoperatively. Prior research indicate that when observation by a geriatric nurse or an occupational therapist is the reference, close relatives tend to under-estimate the patient’s functional performance, cognitively impaired patients tend to overestimate it, whereas cognitively well functioning patients tend to give fairly accurate reports [49-51]. In order to minimise variance, and since a high number of our patients will be cognitively impaired, we do the ADL scores bases on proxy information from relatives or nursing home staff in all the patients.

· Score of the Short Physical Performance Battery (SPPB), a simple test of mobility that can be scored in approximately five minutes and adds up to a sumscore of 0–16 [52]. This score is also primary endpoint in the similar RCT going on in Trondheim, Norway [53]. We have agreed with the Trondheim group to register this endpoint in exactly the same way as they do, thus facilitating pooling of results from the two trials. SPPB will be scored at 4 and 12 months.

### Background variables

The following variables will be registered for descriptive purposes and in order to assess balance between the groups with respect to prognosis:

· Demographics

· Charlson Comorbidity Index [54] and ASA score [55]

· APACHE score [56] and SAPS II [57] for physiological disruption on admittance

· IQCODE score (by close relative or staff in nursing home or home nursing) for pre-fracture cognitive decline

· CDR score (based on the combination of assessment of the patient and interview of a carer)

· Cornell scale for depression in dementia [58]

· Hand grip strength (hand dynamometry) postoperatively and after 4 and 12 months.

· Pre-fracture independence in ADL (Barthel Index and NEADL scored by close relative or nursing home staff)

· Medication

· Body Mass Index

· Type of fracture; surgical and anaesthetic procedures.

### Surveillance of the intervention

Whether or not the intervention turns out to have effect upon the primary outcome, it will be important to establish hypotheses upon why the intervention was (or was not) effective. In multi-component interventions like a hip unit, one single active part of the intervention can normally not be identified. The content of the hip unit intervention is described in detail in the electronic handbook library (see section Intervention above). Due to resource constraints, we are not able to check out in detail to what degree the guidelines are followed. However, we believe that mobilisation and nutritional optimisation may be particularly important components of the complex intervention. Accordingly, we have chosen mobilisation and nutrition as areas of special interest for process monitoring.

Mobilisation during the hospital stay is measured with the small body-warn accelerometer-based sensor ActivPal® [59]. This registration, starting in September 2011, will give objective data as to whether there are differences between the two wards with respect to mobilisation.

Whether or not the nutritional routines are effective is assessed through the registration of weight and weight changes, and through the analysis of micronutrients in blood (Vitamin A, Thiamine, Pyridoxine, Folate, Vitamin B_12_, Vitamin C, 25-Hydroxy-Vitamin D, Vitamin E, Vitamin K, Homocystein, and Zink) four months after the fracture. A nutritionist (AT) is also engaged as a PhD-student working in particular with the nutritional variables.

### Consent and enrolment

Patients are randomised in the Emergency Department in accordance with procedures already established for a recently finished trial [60]. The orthopaedic resident on call assesses all patients admitted for a suspected hip fracture, decide upon the choice of operative treatment, obtain informed consent/assent, and perform the randomisation.

Cognitively intact patients are included on the basis of written, informed consent. We have developed a full information leaflet for cognitively intact patients and a simplified and shortened version. If the latter version is used, a close relative should receive the full version of the patient information. Those who are totally unable to give a valid informed consent will be included on the basis on presumed consent in combination of assent from the nearest relative. The relative is asked for assent at the earliest possible occasion. If a patient that has been assessed as unable to give an informed consent becomes lucid again, he or she is asked to give an own informed consent. If the relative of a patient included on the basis of presumed consent (or the patient him/herself after having become lucid) resists inclusion, all data from that patient will be destroyed.

A large proportion of hip fracture patients are either demented or delirious (or both) on admission. These groups are presumed to be more vulnerable to the quality of hospital care than those that are cognitively intact, and thus important to include in studies of this kind. We have in a previous randomised trial of femoral neck fractures [60] included patients without the ability to give an informed consent, following guidelines from the ethical committee. In that study, 23% of the patients (10% of those admitted from their own homes) were judged by the recruiting surgeon in the Emergency ward to be unable to give an informed consent, either due to dementia or delirium. We have been careful to establish routines for inclusion that take care of these people's dignity and rights, and stay in a close dialogue with respect to this with the Regional Committee for Ethics in Medical and Health Research and the Data Protection Officer.

### Randomisation and allocation concealment

The randomisation is based on computer-generated random numbers, and carried out by a statistician (ES) without any contact with the patients or the personnel involved in the inclusion. We use block randomisation (blocks of variable and unknown size) to ensure an equal group size. The randomisation is stratified with respect to whether or not the patient was admitted from a nursing home, in order to get the groups balanced regarding pre-fracture cognitive decline, an important prognostic factor. The allocation of each patient (orthogeriatric or orthopaedic care) is by sealed, opaque, numbered envelopes that are held in the Emergency Department (different colours for the two stratification groups). For each randomised patient, the study physician (LOW) checks that the randomisation envelope with the lowest number has been used.

The study physician (LOW) and the study nurses monitor all patient admissions for hip fracture at the hospital. Eligible patients that were not included are registered, with the reason for non-inclusion. For patients who erroneously have not been included but are eligible and for whom a valid consent or assent is available, we accept randomisation if the patient can be moved to the ward he or she becomes randomised to immediately after having arrived to the orthopaedic ward. If the error is detected later on, the patient is registered as lost from inclusion.

All variables collected at the 4 and 12 months follow-up are collected by study nurses blind to allocation. The study nurses register cases in which they have been unblinded, for instance because patients or relatives have disclosed the allocation. This has happened in 5-10% of the cases (varying a bit between the different nurses). The research personnel caring for the patients during the stay cannot be blinded, meaning in-hospital variables are not acquired blind to allocation.

### Data collection

Background information, information regarding surgical and anaesthetic procedures, drug use, and proxy information on pre fracture ADL function and cognitive function is collected during the acute stay, by the study physician and a study nurse. On day 1 a research nurse attaches an ActivPal® sensor on the anterior aspect of the non-affected thigh. The sensor is worn for a minimum of 72 hours or until discharge or until it has to be removed through patient action or choice.

Research assistants continually scrutinise study forms for missing data. Missing data from proxies are collected through telephone calls. Electronic hospital records give further information on clinical examinations, medication, blood tests and other investigations performed during the index stay.

The 4-month registration is performed by a research assistant at the site where the patient is living, irrespective of location. This might be the patient's own home, a nursing facility or a rehabilitation institution. The time window is 4 months ± 3 weeks. 

The 12-month registration is performed similar to the 4-month registration. The time window is 12 months ± 3 weeks. For details on data collection and questionnaires, see Table 1.

**Table 1:** Variables and time-points for assessment

**Pre-fracture characteristics registered during index stay**	**Index stay**	**4-months follow-up**	**12-months follow-up**
IQCODE	Demographics	CERAD 10 words memory task	CERAD 10 words memory task
CDR	Type of fracture	MMSE	MMSE
Barthel	Surgical and anaesthetic procedures	Cock drawing test	Cock drawing test
NEADL	Medication	CDR	CDR
Cornell	CCI	Barthel	Barthel
	ASA score	NEADL	NEADL
	APACHE score	Cornell	Cornell
	SAPS II	SPPB	SPPB
	ECG	Weight	Weight
	Medication	Hand-dynamometry Micronutrients in blood (as during index stay)	Hand-dynamometry Micronutrients in blood (as during index stay)
	Hand-dynamometry	Markers of bone turnover (as during index stay)	Markers of bone turnover (as during index stay)
	Weight, height		
	CAM daily		
	MDAS		
	ActivePAL (from September 2011)		
	Micronutrients in blood (Vit A, Thiamine, Pyridoxine, Folate, Vit B_12_, Vit C, 25-Hydroxy-Vit D, Vit E, Vit K_1_, Homocystein, Zink)		
	Markers of bone turnover (Osteocalcin, Undercarboxylated osteocalcin, Parathyroid hormone, Bone-specific alkaline phosphatase, Insulin-like growth factor 1, Carboxy-terminal collagen crosslinks (CTX), Aminoterminal propeptide of type I collagen (PINP))		

Abbreviations: ALP = Alkaline Phosphatase. APACHE = Acute Physiology And Chronic Health Evaluation. ASA = American Society of Anaesthesiologists. CAM = Confusion Assessment Method. CCI = Charlson Co-morbidity Index. CDR = Clinical Dementia Rating. ECG = electrocardiogram. IGF = Insuline-like Growth Factor. IQCODE = Informant Questionnaire on Cognitive Decline in the Elderly. MDAS = Memorial Delirium Assessment Scale. NEADL = Notingham Extended Activities of Daily Living Scale. SAPS = Simplified Acute Physiology Score. SPPB = Short Physical Performance Battery. Vit = Vitamin.

### Data analyses and statistical power

The primary analyses will be done in a modified intention-to-treat analysis including the sample of patients completing the CERAD 10 words test and CDR at 4 months. The weighting of the components of the primary endpoint (see primary endpoint section above) will be definitely decided upon before any analyses of the treatment effect are initiated. A separate per protocol analysis will be undertaken. The primary endpoint will be analysed by stratified linear regression. If the assumption of normality turns out to be violated, additional analyses will be performed on transformed data as well as by non-parametric methods (Mann–Whitney test). Binary outcomes will be analysed by chi square tests and logistic regression models. The material will be checked for any inequality in the distribution of important prognostic variables between the two arms, and if such are present, they will be adjusted for by including these in appropriate regression models.

Sensitivity analyses will be performed by imputing missing values in different conservative ways, e.g. by imputing the worst observed score of the treatment group and also by random sampling from the observed scores in the treatment group.

The main analyses of 12 months data will be carried out including patients with responses registered at that time point. Additional sensitivity analyses will be performed in the sample of all randomised patients using different methods of imputation as described above as well as by carrying the four month observation forward.

If an interaction test is statistically significant at the 0.10 level, we will analyse the effect upon the endpoints separately in the following subgroups: patients admitted from nursing homes or not, and patients with and without pre-fracture dementia.

We also register the time window from admittance to start of surgery, as any group difference here may also impact severely on the outcome [7].

No data are available allowing us to carry out precise power estimates based on our primary endpoint. Based upon previous experiences with CDR, however [61] (Engedal, unpublished data), we judge 300 patients to be sufficient to detect clinically meaningful differences. As 20% of hip fracture patients can be expected to die before the 4 months follow up [6], we aim for 370 patients to be randomised.

The results of the trial will be published in accordance with the CONSORT guidelines [62]. Publishing the protocol of the Oslo Orthogeriatric Trial is in accordance with the recommendation to publish RCT protocols for complex interventions [63].

### Time plan of the study

**Figure 1:**
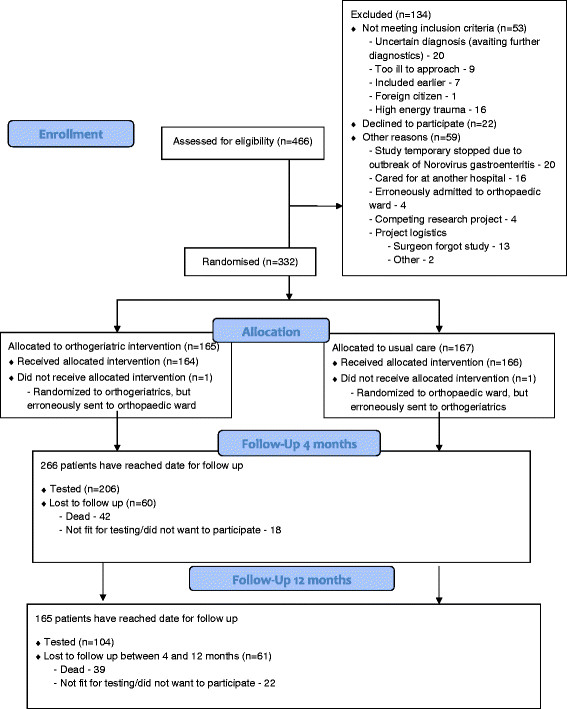
Patient flow, updated by January 7th, 2012.

The first patient was randomised on 17^th^ September 2009, and inclusion was closed at 5th of January 2012. 332 patients have been randomised. In the same period, 466 patients have been admitted to the hospital with a suspected hip fracture (see Figure 1). The last patient will reach the 4-months registration in May 2012 and the 12-months registration in January 2013.

The formal effect analyses are estimated to start in the summer or autumn of 2012, when all the 4-months data have been entered and checked. Analysis of secondary outcomes based upon the 12-months registration can start in the spring of 2013.

### Ethics and approvals

The main ethical grounds for the study is based upon the uncertainty principle; at present we have insufficient evidence as to know which organisational model is the best one for hip fracture patients. The research literature give some reasons to believe that an orthogeriatric approach will be superior, but such a model may also introduce pitfalls, for instance that the patients will have access to too weak orthopaedic competence. When organisational and economical constraints force us to divide the treatment of this patient group between two departments, we find a randomised allocation (enabling us to provide better evidence within this field) to be more ethical as any other way of allocating patients between the two wards.

The study is approved by the Regional Committee for Ethics in Medical and Health Research (South-East Norway) (REK S-09169a) and the Data Protection Officer at Oslo University Hospital (Ref. 1361).

## Discussion

The main focus of the Oslo Orthogeriatrics Trial is on cognition, in particular delirium prevention and thereby potentially beneficial long-term cognitive effects of orthogeriatric care. We have chosen this primary outcome because delirium is extremely common in hip fracture patients [7], there is increasing evidence that delirium may have long-term negative consequences upon cognition [9,10], and such consequences are devastating.

Our choice of primary outcome rests upon two important pre-suppositions, and may thus be regarded as audacious. First, orthogeriatric care must result in a decreased incidence of delirium in the acute phase, and, secondly, delirium prevention must result in better cognition that can be measured after 4 months. If the trial should turn out to be negative, any of these two presuppositions may be false. Since we measure the incidence of perioperative delirium, we may be able to draw some inference regarding the proposed order of causation even if the study should be negative, though we cannot completely rule out the possibility that the registration of delirium is more complete in the intervention group due to higher sensitivity for this phenomenon among the nurses and thus more precise reports to the study staff. Other potential weaknesses are insufficient sensitivity in the outcome measure, the risk of a type 2 error from an insufficient sample population. Moreover, the orthopaedic department at our hospital has for many years had a particular interest in hip fracture patients, and the orthopaedic nurses are very dedicated to the care and mobilisation of these patients. Accordingly, "usual care" in our experiment may well be of higher quality than the average of orthopaedic departments, decreasing the possibility of finding any further benefit of the orthogeriatric model. With respect to quality of the service, we register some aspects of mobilisation and nutrition, as well as pre-operative waiting time, as explained above. Our choice of supplementary outcome variables reflecting functioning in a broader sense, e.g. mobility, ADL and place of residence, will enable us to shed light on other potential effects of an orthogeriatric service. However, if the main outcome should be negative, any significant effects on some of the secondary outcomes will certainly be hypothesis generating.

Since the focus of the trial is cognition and prevention of cognitive decline, we have defined wider eligibility criteria than many other hip fracture trials. Nursing home residents and other patients with pronounced frailty are particularly vulnerable to sub-optimal clinical quality, and are at high risk of developing dementia and of worsening of a pre-existing dementia. Accordingly, these patients are included in our study, even though they have a very limited rehabilitation potential with respect to for instance return to own home or independent mobility. Likewise, we have not set a lower age limit for our study, but exclude those who fracture their hip due to a high energy trauma. Nearly every hip fracture patient is above 70 years, but, in our opinion, a hip fracture due to a low energy trauma in a person below 70 is an indicator of frailty, making inclusion in this trial logical. The same reasoning applies to those with pathological fractures, for instance due to malignancy. These patients are also prone to delirium, and thus should be eligible for our study regardless of their potential to achieve independent gait function, unless their life expectancy is very short. Our wide inclusion criteria imply a good external validity, but at the expense of an increased heterogeneity in the patient sample.

It follows from the inclusion criteria that our participants will have very different levels of cognitive performance, from those with severe dementia to those who function well. Our hypothesis is that optimal and integrated care may help to preserve cognitive function on the entire range of pre-fracture functioning. This assumption makes the choice of primary endpoint challenging. We decided to use a composite endpoint consisting of immediate and delayed recall from the 10 word memory list and the CDR, as we believe that the first instrument will be able to capture differences between cognitively well functioning participants, and the latter one differences in the lower part of the functional spectrum. Composite endpoints in randomised trials are sometimes regarded as controversial [64,65]. The critique is, however, mostly related to the situation where the elements of the composite are of different importance, and in particular when any group difference is most likely to be driven by the less important part. The same kind of reasoning is appropriate for dementia research, where an individual quantification of impairment, disability and handicap is recommended [66]. However, in our study the composite endpoint does not reflect different domains, but aims at measuring the same construct, i.e. cognitive functioning. We consider the elements to be equally important, but we consider one single scale to have insufficient sensitivity to catch every relevant change. Moreover we anticipate the variability within each scale to be too high, so that it will be impossible to catch clinically meaningful differences with reasonable power by the use of one single scale. The normalising of each scale to a 0–100 scaling is in accordance with established practice within quality of life research [67], and the use of CDR as a global measure of cognitive functioning is in accordance with official recommendations [66]. We will certainly report the results also for each element of the composite, as generally recommended for studies utilising composite endpoints [64,65].

Clinically, our model is characterised by the strong emphasis on preoperative geriatric care, and the fact that the intervention is developed within the frame of an ordinary acute geriatric ward treating frail patients with acute general medical conditions like infections, acute heart diseases, pulmonary embolism, obstructive pulmonary disease and so on. As such patients are also prone to develop delirium, measures for delirium prevention are relevant for the ward's general geriatric patients as well as for the hip fracture patients. A potential drawback is, however, that this patient mix makes it more difficult to build competence specifically for the hip fracture group.

Our intervention does not include any geriatric follow-up after the index stay. In this phase, the patients are offered care as usual by the primary health service. Intervention as well as control patients are offered follow-up at the orthopaedic out-patient clinic in accordance with that department’s routines. The lack of geriatric follow-up may decrease the chance of finding an effect of the intervention. On the other hand, our focus on delirium prevention as a means to prevent further cognitive decline, makes it logical to put emphasis on the immediate peri-operative phase where delirium is extremely common.

Our study differs in certain ways from other ongoing hip unit trials, for instance the Trondheim Hip Fracture Trial, with whom we are collaborating [53]. While our trial focuses on delirium prevention and cognition, the Trondheim trial focuses upon mobility. Thus, the two trials may provide knowledge that is complimentary regarding differential effects of orthogeriatric care. Our choice of a secondary endpoint (SPPB) that is similar to the primary endpoint in the Trondheim trial allows us, however, to pool our data with respect to this outcome. As the interventions in the two trials are very similar, such an aggregation of data is, in our opinion, well justified.

It is our hope that the Oslo Orthogeriatrics Trial will help broadening the evidence base for optimal organising of services for hip fracture patients. Through our choice of intervention emphasis (preoperative and early postoperative intervention) as well as outcome (cognition), we will be able to shed light on which elements of an orthogeriatric care that is most relevant as well as which aspects of the patient outcome upon which such an intervention may have an impact [13].

## Abbreviations

ADL: Activities of Daily Living; ASA: American Association of Anaesthesiologists; CAM: Confusion Assessment Method; CDR: Clinical Dementia Rating; CERAD: Consortium to Establish a Registry for Alzheimer's Disease; CI: Confidence Interval; ICD: International Classification of Diseases; IQCODE: Informant Questionnaire on Cognitive Decline in the Elderly; LOS: Length of Stay; MDAS: Memorial Delirium Assessment Scale; MMSE: Mini Mental State Evaluation; NEADL: Nottingham Extended ADL Scale; RCT: Randomised Controlled Trial; RMO: Refused Missed or Otherways excluded; SAPS: Simplified Acute Physiology Score; SPPB: Short Physical Performance Battery.

## Competing interests

The authors declare that they have no competing interests.

## Authors’ contributions

TBW initiated the study, has led the work on research design, intervention and implementation of the study protocol and is the primary author of the manuscript. LOW has the daily responsibility of running the study and collecting the data. AT has particular responsibility for collecting, analysing and interpreting nutritional data, has designed the nutritional part of the intervention, and has also participated extensively in general planning of the study and in the data collection. KE has participated in all aspects of the project planning, in particular regarding the cognitive endpoints. FF, VJ, IS, JR and SC all made important contributions to the planning of the study and writing of the protocol. ES carried out the randomisation procedure and has participated extensively in planning of the statistical analyses. All authors read and approved the final manuscript.

## Pre-publication history

The pre-publication history for this paper can be accessed here:

http://www.biomedcentral.com/1471-2318/12/36/prepub
